# Fast optical cooling of a nanomechanical cantilever by a dynamical Stark-shift gate

**DOI:** 10.1038/srep14977

**Published:** 2015-10-12

**Authors:** Leilei Yan, Jian-Qi Zhang, Shuo Zhang, Mang Feng

**Affiliations:** 1 State Key Laboratory of Magnetic Resonance and Atomic and Molecular Physics, Wuhan Institute of Physics and Mathematics, Chinese Academy of Sciences, Wuhan, 430071, China; 2 University of the Chinese Academy of Sciences, Beijing 100049, China; 3 Zhengzhou Information Science and Technology Institute, Zhengzhou, 450004, China

## Abstract

The efficient cooling of nanomechanical resonators is essential to exploration of quantum properties of the macroscopic or mesoscopic systems. We propose such a laser-cooling scheme for a nanomechanical cantilever, which works even for the low-frequency mechanical mode and under weak cooling lasers. The cantilever is coupled by a diamond nitrogen-vacancy center under a strong magnetic field gradient and the cooling is assisted by a dynamical Stark-shift gate. Our scheme can effectively enhance the desired cooling efficiency by avoiding the off-resonant and undesired carrier transitions, and thereby cool the cantilever down to the vicinity of the vibrational ground state in a fast fashion.

Over the past years, nano-mechanical resonators (NRs) have attracted considerable attention both theoretically and experimentally and presented potential applications based on the quantum properties, for example, optomechanical induced transparency^1^, photon blockade^2^,^3^, optical Kerr effect^4^, entanglement between microscopic objects^5^,^6^, quantum state measurement^7^,^8^, biological sensing detection^9^,^10^ and hybrid coupling to cold atoms^11^.

However, quantum properties regarding the NRs are always hidden by the thermal phonons involved. To suppress the thermal phonons, many schemes have been proposed so far to try to cool the NRs down to the vicinity of their vibrational ground states, such as the sideband cooling^12^,^13^, the backaction sideband cooling^14^,^15^,^16^,^17^,^18^,^19^, the hot-thermal-light-assisted cooling^20^, the time-dependent control cooling^21^,^22^, the quadratic-coupling-based cooling^23^, the measurement cooling^24^ and the electromagnetically induced transparency (EIT) cooling^25^,^26^,^27^,^28^,^29^.

The EIT cooling works based on quantum interference, which enhances the first-order red-sideband transition for cooling, but eliminates the carrier transition and suppresses the first-order blue-sideband transition for heating^25^,^26^,^27^,^28^,^29^. In particular, it works efficiently even in the non-resolved sideband regime in the laboratory representation, i.e., with a large spontaneous emission rate. The EIT cooling was first proposed and experimentally implemented in the trapped-ion system^30^,^31^, and then extended to other systems, including the quantum dot^25^,^27^,^28^, the superconducting flux qubit^26^ and the diamond nitrogen-vacancy (NV) center^29^. However, for the Rabi frequency comparable to the vibrational frequency of the NR, the prerequisite of the fast cooling, the existing cooling scheme could not work efficiently^29^. Therefore, developing an alternative scheme available for the NR cooling, which is faster and more efficient than the EIT cooling, is highly desirable^32^. On the other hand, a NV center coupled to a nanomechanical cantilever can be used to cool the cantilever vibration down to a quantum regime^29^,^33^, where the coupling is from a magnetic field gradient (MFG). The extension of such a coupling is applicable to future scalable quantum information processor^33^,^34^. To this end, achievement of high-quality NRs and cooling low-frequency NRs are highly expected, but experimentally demanding.

The present work focuses on the ground-state cooling of a NR with the assistance from a Stark-shift gate in the non-resolved sideband regime in the laboratory representation. Such a cooling scheme can cool a low-frequency (≤1 MHz) NR more efficiently than the conventional sideband cooling due to elimination of the involved carrier transitions which contribute for heating, as confirmed in^32^ for cooling the trapped ion. However, compared to the trapped ion, the NR (i.e., the cantilever) under consideration is of a much bigger mass, which decreases the mechanical effect of light on the NV-cantilever to nearly zero. To generate a strong enough coupling between the NV center and the cantilever, we introduce a strong MFG, as discussed in^29^. Moreover, since the cantilever is more sensitive to the environmental noise than the trapped ion, we have to seriously consider the influence from the non-zero temperature thermal noise of the environment in our calculation.

The key point in the present work is the introduction of an effective classical field to couple the sublevels of the electronic ground state of the NV center, which creates a dynamical Stark shift under the strong MFG and accelerates the cooling of the cantilever by suppressing the undesired transitions. We show the possibility to cool the cantilever with the same cooling rate as in the trapped-ion system^32^. Moreover, different from the microwave cooling scheme^33^, in which the magnetic tip with a fixed MFG is attached at the end of the cantilever, the cooling in our case is made by lasers, which ensure that the cooling rate (the cooling speed) in our scheme is larger (faster) than in the previous scheme^33^, and the MFG in our idea is independent from the cantilever, but generated by the coils and controlled by the external electric current. The MFG in our design is evidently more flexibly adjustable.

More specifically, we show below that the addition of the Stark-shift gate makes the cooling more powerful than the optics-based EIT cooling in a previous scheme^29^, and is particularly useful for the cantilever of lower vibrational frequency under weaker laser irradiation. This is much different from the strong coupling condition required in previous EIT-like schemes using cavities^35^,^36^. Since the cooling of the low-frequency cantilevers down to the ground state is still challenging with current technology, and the requirement of weak laser irradiation can reduce the experimental difficulty, our scheme is of practical application in exploring quantum properties of the nanomechanical cantilevers.

## Results

### The cooling of a NV-cantilever system by a Stark-shift gate

**Figure 1:**
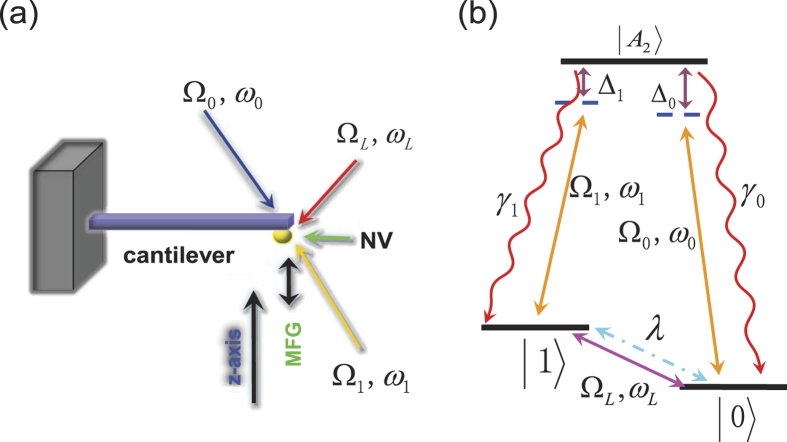
(**a**) Schematic illustration of our cooling scheme using a dynamic Stark-shift gate, where the nanomechanical cantilever is attached by a NV center under irradiation of lasers and an effective classical field. (**b**) The three levels form our major part of the cooling, where the irradiation of the two lasers satisfies the two-photon resonance with Δ_0_ = Δ_1_ and the effective classical field is in resonance with the transition between the two ground states, i.e., *ω*_*L*_ = *ω*_0_ − *ω*_1_. The cantilever vibration is coupled to the NV center by a strong MFG. *γ*_0_ (*γ*_1_) is the decay from the excited state 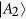 to the ground state 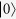 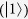.

Our system is modeled as in Fig. 1(a), where a negatively charged NV center is attached at the end of a nanomechanical cantilever under a strong MFG. The ground state of the NV center is a spin triplet with a zero-field splitting 2*π* × 2.87 GHz between *m*_*s*_ = 0 and *m*_*s*_ = ±1, where *m*_*s*_ is the projection of the total electron spin *S* = 1 along the *z*-axis. The sublevels *m*_*s*_ = ±1 are employed for qubit encoding in our cooling scheme, with *m*_*s*_ = −1 as 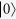 and *m*_*s*_ = +1 as 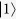. According to the selection rule of the transitions^37^,^38^, the state 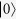 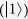 may be coupled to the excited state 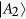 by a polarized laser^29^,^37^,^38^,^39^. 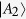 is an entangled state involving the components 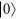, 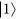 and the orbital states, and keeps separate enough from neighboring levels^38^. The state 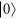 can be coupled to 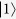 by an effective classical field due to two-photon Raman process (Adopted in the present work; see Methods for details) or by a stress applied perpendicularly to the axial direction of the NV center^40^. In Fig. 1(b), the 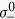 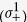 polarized laser owns the frequency *ω*_0_ (*ω*_1_) and the Rabi frequency Ω_0_ (Ω_1_). The effective classical field is with the frequency *ω*_*L*_ and the Rabi frequency Ω_*L*_.

It should be noted that there are leakages from the excited state 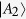 down to the metastable state 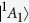 (not shown in Fig. 1(b)), which would stop the cooling process. To solve this problem, we have to employ *m*_*s*_ = 0 and other auxiliary states to recycle the cooling process, as discussed in^29^ but not reiterated in the present paper. Furthermore, different from the trapped-ion system, in which the coupling between the internal and the vibrational degrees of freedom is caused by the mechanical effect of light^41^, our model employs the MFG to provide the coupling between the NV center and the vibration of the cantilever. The MFG consists of a coil wrapping a permanent magnet core, controlled by the external electric current.

The Hamiltonian of the system in units of *ħ* = 1 is given by


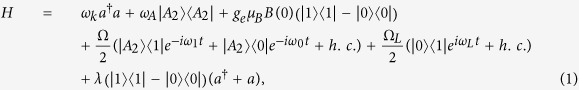


where *a*^†^ (*a*) is the creation (annihilation) operator of the cantilever vibration with frequency *ω*_*k*_, *ω*_*A*_ is regarding 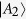, *B*(0) is the constant magnetic field strength, *g*_*e*_ is the *g*-factor and *μ*_*B*_ is the Bohr magneton. Ω( = Ω_0_ = Ω_1_) and Ω_*L*_ are the Rabi frequencies regarding irradiation from the laser and the effective classical field. *λ* = *g*_*e*_*μ*_*B*_*B*′(0)*x*_0_ is the coupling due to the MFG *B*′(0) with 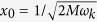 and a cantilever mass *M*^33^,^34^,^42^. Although the NV center is sensitive to the strain, we suppose a low strain condition throughout this work, which ensures that the employed excited state 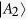 robustly owns stable symmetry properties^38^.

To understand the cooling physical picture and simplify the calculation, we make a unitary transformation on equation (1). In the rotating frame, we have 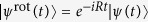 and *H*^rot^ = *e*^−*iRt*^*H*(*t*)*e*^*iRt*^ + *R* with 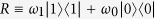
43. Under the near-resonance condition *ω*_*L*_ ≈ *ω*_0_ − *ω*_1_, equation (1) can be rewritten in a time-independent form as *H*^rot^ = *H*_0_ + *V*, with


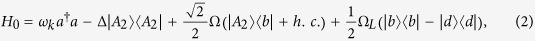


and


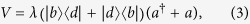


where 
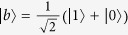
 and 
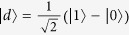
 are the corresponding bright and dark states, respectively, and the detunings satisfy Δ ≡  Δ_0_ = Δ_1_ with Δ_0_ = *ω*_0_ − *ω*_*A*_ − *g*_*e*_*μ*_*B*_*B*(0) and Δ_1_ = *ω*_1_ − *ω*_*A*_ + *g*_*e*_*μ*_*B*_*B*(0). Moreover, the last term in equation (2) describes the energy difference between the bright and dark states caused by the effective classical field for the Stark shift, by which a Stark-shift gate will be performed below for the cooling of the cantilever vibration. Besides, the coupling between the cantilever and the NV center is created by a strong MFG, which makes the first-order red-sideband transition dominate in the cooling process based on quantum interference. With assistance of the effective classical field, the energy difference between the dark and bright states is equal to the frequency of the cantilever vibration. As a result, the phonon is dissipated by the coupling due to the MFG with the assistance of external fields.

### The cooling mechanics based on the Stark-shift gate

The cooling in our scheme is based on the Stark-shift gate. According to refs 32,42,43, the Stark-shift gate in the total Hamiltonian *H*_0_ + *V* is described by


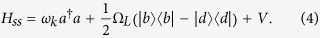


In the interaction picture, after the rotating wave approximation is applied, the Hamiltonian in (4) can be rewritten as


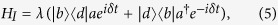


with *δ* = Ω_*L*_ − *ω*_*k*_. When our model is operated at the work point of the Stark-shift gate (Ω_*L*_ = *ω*_*k*_), the above Hamiltonain reduces to


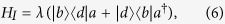


which is a typical Jaynes-Cummings interaction for the first-order red-sideband transition between the dark and bright states, leading to the phonon number change in the cantilever vibration.

**Figure 2:**
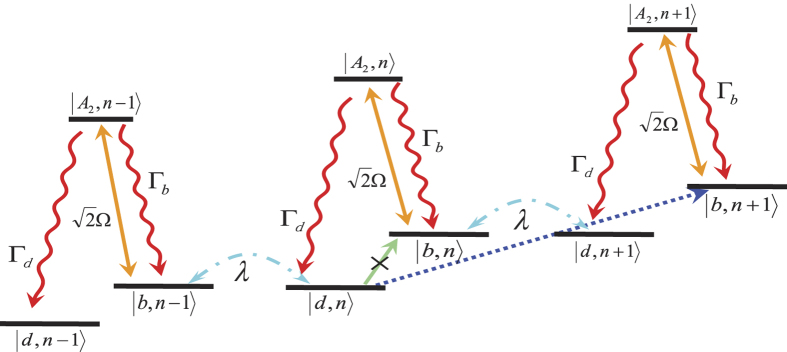
The schematic for the cooling mechanism. The Stark-shift-gate assisted cooling includes an effective classical field creating the Stark shift of the ground state, a strong MFG whose coupling *λ* leads to the red-sideband transition 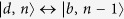, and the optical lasers couple the bright state 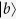 to the excited state 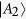. The optical lasers should satisfy the two-phonon resonance condition (Δ_0_ = Δ_1_), which yields elimination of the carrier transition between 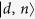 and 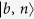 and suppression of the blue-sideband transition between 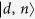 and 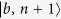. So the strong MFG only contributes to the red-sideband transition. In our treatment, we suppose Γ_*d*_ = Γ_*b*_ = Γ/2 and Γ = *γ*_1_ + *γ*_2_, and other parameters are defined in the text or in Fig. 1.

The cooling process in our scheme is described in Fig. 2. In terms of equation (6), if the system is initially in the state 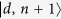, the only possible transition is from 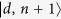 to 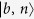, which is caused by the Stark-shift gate. It results from the fact that the blue-sideband transition and the carrier transition relevant to the dark state 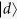 are suppressed by quantum interference. In the laboratory frame, the transition 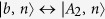 is actually driven by two lasers with the same Rabi frequencies and detunings. As it is plotted in Fig. 2, the spin state is first excited to 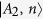, and then decays to the bright state 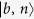 or the dark state 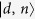. If the decay is to the dark state 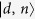, one phonon is lost from the cantilever vibration due to *H*_*I*_ and the cooling goes to the next step. But if the decay is to the bright state 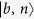, the state will be pumped to the excited state 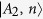 again, and this cycle of the laser cooling will be repeated until the decay is to the dark state.

A clearer picture for above cooling process with the phonon dissipation governed by the transition 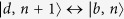 can be found in Supplementary Information by numerically calculating the fluctuation spectra. We may find that the carrier transition 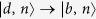 is totally suppressed by the destructive interference, and the blue-sideband transition 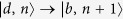 is largely suppressed. As a result, repeating the laser cooling cycles, we will finally cool the cantilever down to the vibration ground state.

Before going further to the numerical calculation, we simply compare the Stark-shift-gate cooling with the EIT cooling in ref. 29. From the schematic illustrations, both of them share the similar quantum interference process and steady state, which can effectively suppress the blue-sideband transition and the carrier transition. Besides, the Stark-shift-gate cooling goes beyond the EIT cooling by an additional coupling, which drives the transition between the dark and bright states, constituting a Jaynes-Cummings interaction by the first-order red-sideband transition at the work point Ω_*L*_ = *ω*_*k*_. As a result, different from the EIT cooling, the Stark-shift-gate cooling works with the efficiency independent from the cooling laser strength, but mainly relevant to the work point.

### The analytical and numerical treatments for the cooling

By utilizing the perturbation theory and the non-equilibrium fluctuation-dissipation relation, the Hamiltonian *H*^*rot*^ (equation (2) plus equation (3)) yields the heating (cooling) coefficient *A*_+_ (*A*_−_) caused by the external fields as below,


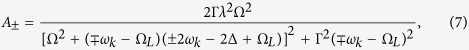


whose deduction in details can be found in Methods and Supplementary Information. Γ is the total decay rate regarding 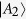. The heating (cooling) coefficient in equation (7) is different from the one obtained previously^26^,^29^,^30^,^44^, but can be reduced to the result in^32^ when Ω_*L*_ = *ω*_*k*_. This is due to the fact that both^32^ and our scheme share the same work point for the Stark-shift gate, related to the Rabi frequency of the effective classical field and the vibrational frequency of the ion or cantilever. Nevertheless, the cooling (heating) efficiency in^32^ only depends on the Rabi frequency of the microwave field, but ours is relevant to the effective classical field Rabi frequency assisted by the MFG coupling *λ*.

**Figure 3:**
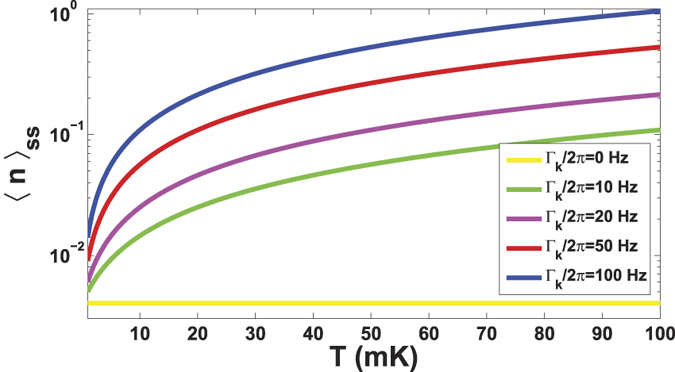
The final phonon number 〈*n*〉_*ss*_ (in logarithmic scale) versus the environmental temperature *T* and the vibrational decay rate Γ_*k*_, where we have taken the parameter values *ω*_*k*_/2*π* = 2 MHz, Ω/2*π* = 2 MHz, Ω_*L*_ = *ω*_*k*_ and Δ = 0^37^, as well as Γ/2*π* = 15 MHz and *λ*/2*π*  = 0.115 MHz^33^,^34^,^50^.

A proper analysis of the phonon dissipation must consider the non-zero temperature environmental noise, since compared to the trapped-ion system the cantilever with much larger volume and mass is more sensitive to the environment. Using the techniques developed previously^26^,^29^, we obtain following analytical expression of the time-dependent average phonon number,


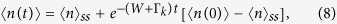


where the cooling rate *W* = *A*_−_ − *A*_+_ originates from the interaction between the NV center and the external fields^30^,^44^. The final average phonon number is


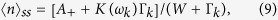


where Γ_*k*_  ≡  *ω*_*k*_/*q* is the vibrational decay rate with the quality factor *q* of the cantilever, and 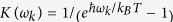 is the thermal occupation for the cantilever vibrational degrees of freedom^26^ with the Boltzmann constant *k*_*B*_ and the environmental temperature *T*, respectively. As plotted in Fig. 3, our scheme works even for the NR with a low frequency (e.g., *ω*_*k*_/2*π* = 2 MHz). Compared with the previous scheme^29^, our scheme can achieve a good cooling under very weak cooling laser radiation (Ω/2*π* = 2 MHz). With the same environmental temperature *T*, our scheme works for a smaller decay rate Γ_*k*_. More specific discussion on this point can be found later.

For a deeper understanding of our cooling scheme, we may focus on the work point Ω_*L*_ = *ω*_*k*_ of the Stark-shift gate, which simplifies the heating and cooling coefficients in equation (7) to be


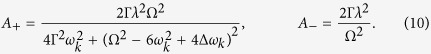


**Figure 4:**
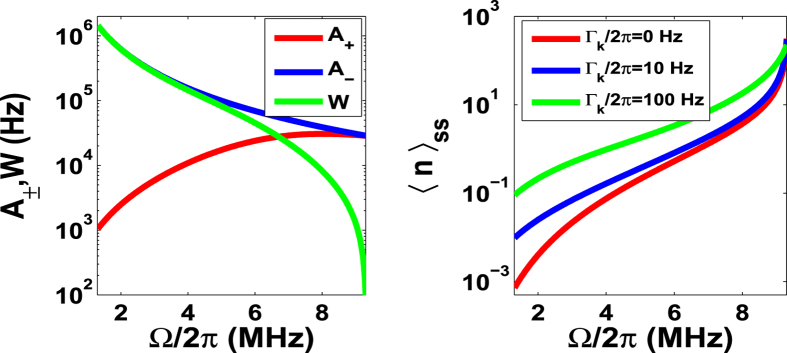
(Left) The heating coefficient *A*_+_, the cooling coefficient *A*_−_, and the cooling rate *W* = *A*_−_ − *A*_+_ (in logarithmic scale) versus Ω/2*π*. (Right) The final average phonon number 〈*n*〉_*ss*_ (in logarithmic scale) versus Ω/2*π* for different Γ_*k*_, where we consider Ω_*L*_ = *ω*_*k*_ and the other parameters take the values as *ω*_*k*_/2*π* = 2 MHz, Γ/2*π* = 15 MHz, *T* = 20 mK, *λ*/2*π* = 0.115 MHz and Δ = 0.

As plotted in Fig. 4(Left), *A*_+_ (*A*_−_) increases (decreases) with Ω. To make sure an efficient cooling, we should have *A*_−_ to be larger than *A*_+_, implying an upper limit 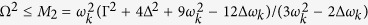 from the above analytical expressions. Moreover, both the left and right panels of Fig. 4 show that the faster cooling and the minimal final phonon number prefer smaller laser Rabi frequency. The extreme case happens at Ω = 0, in which we have *W* = *A*_−_ due to negligible *A*_ + _, and thereby 〈*n*〉_*ss*_ tends to minimum. However, this is a non-physical condition since Ω = 0 means no laser irradiation. In our case, if the cooling works, Ω^2^ > *M*_1_ = max [Γ*λ, ω*_*k*_*λ*, Δ*λ*] (meaning the internal dynamics faster than the external dynamics, e.g., 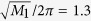 MHz in Fig. 4) should be satisfied. As such, we reach a trade-off regime for the laser irradiation *M*_1_ < Ω^2^ ≤ *M*_2_. On the other hand, the laser detuning Δ involved in *A*_+_ also has influence on the cooling. To have a larger cooling rate, a larger blue detuning (i.e., Δ > 0) is required for the condition 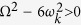, while a larger red detuning (Δ < 0) is necessary when 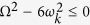 is satisfied.

**Figure 5:**
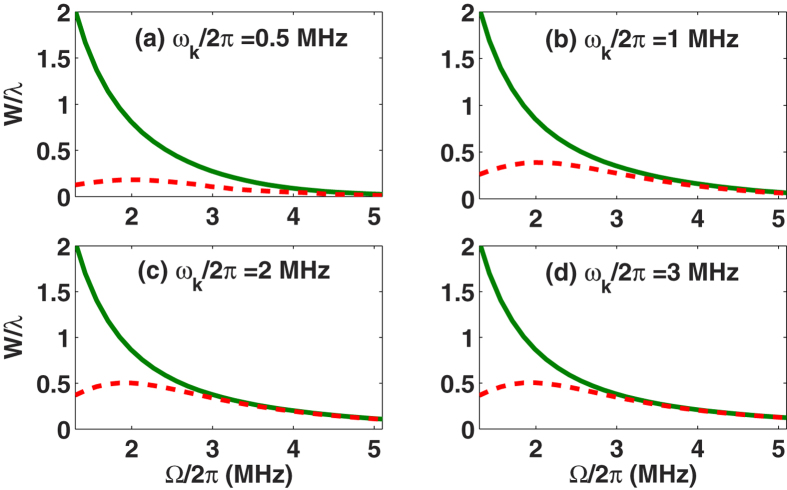
The cooling rate *W*/*λ* versus the Rabi frequency Ω/2*π* where the work point Ω_*L*_ = *ω*_*k*_ is satisfied. The parameters take values as *λ*/2*π* = 0.115 MHz, Γ/2*π* = 15 MHz and Δ = 0. The solid curves are the analytical results of the cooling rate *W* by equation (10) and the dashed curves are plotted by solving the master equations of *H*^*rot*^.

The analytical results above (i.e., equations (7) and (10)) are obtained under the perturbation and the adiabatic condition. This implies that the real cooling effect should be justified by the small values of Ω, for which the adiabatic condition is not fully satisfied. To this end, we have numerically calculated the cooling rate *W* with respect to Ω at the work point of the Stark-shift gate. We may find from Fig. 5 that the discrepancy between the analytical and numerical results appears within the regime Ω/2*π* < 3 MHz where the adiabatic condition is no longer valid. If we check this regime more carefully, we find that the discrepancy is bigger for the lower frequency of the cantilever, which is due to the fact that the lower frequency cantilever owning a larger *λ* (*λ* is a function inversely proportional to 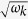) makes the rotating wave approximation less valid in obtaining the work point of the Stark-shift gate.

The physical reason for the cooling rates plotted in Fig. 5 can be understood by the decay and the pumping in the cooling process. Since the transition between the bright and dark states is dipolar forbidden, we excite the system from the bright state 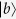 to the excited state 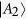, and then it decays down to the dark state 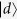. With a stronger pump light, the effective decay from the bright state to the dark state would be bigger, which yields a larger cooling rate. However, a much stronger light would shift the bright state and thereby weaken the red-sideband transition. As a result, with increasing Ω, the cooling rate increases at first, and then decreases, as examined by numerics in Fig. 5. However, the analytical solutions in equation (10) could not exactly describe the above cooling process if Ω/2*π* < 3 MHz.

**Figure 6:**
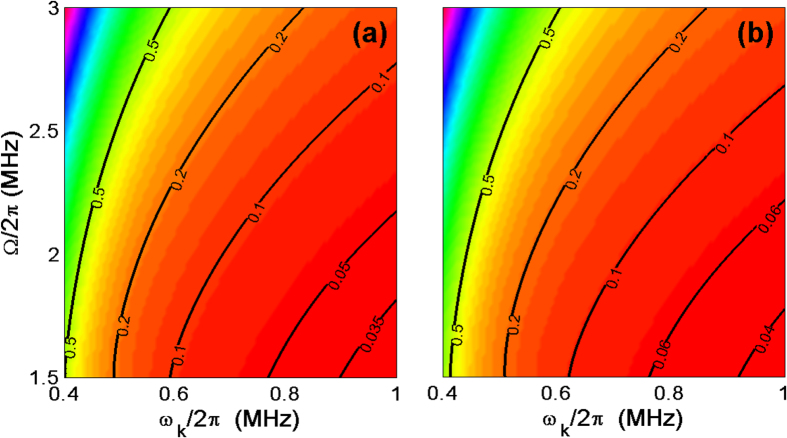
The final average phonon number 〈*n*〉_*ss*_ versus the vibrational frequency *ω*_*k*_/2*π* and Rabi frequency Ω/2*π*, where the work point Ω_*L*_ = *ω*_*k*_ is satisfied. (**a**) The case of zero temperature environment without considering the vibrational decay. (**b**) The case of the environmental temperature *T* = 20 mK with the vibrational decay rate Γ_*k*_/2*π* = 1 Hz. Other parameters employed are *λ*/2*π* = 0.115 MHz, Γ/2*π* = 15 MHz and Δ = 0.

Both the analytical and numerical results imply that our cooling scheme is more powerful than previously proposed ones^25^,^26^,^27^,^28^,^29^, particularly in the case of the lower vibrational frequency (*ω*_*k*_/2*π* < 1 MHz) and the weaker laser field (Ω/2*π* < 3 MHz). Considering the numerical results in Figs 5 and 6, we observe that the maximal cooling rate *W*_*max*_ in our case can be more than 0.5*λ* once *ω*_*k*_/2*π* ≥ 2 MHz, implying a much better cooling than in the resolved sideband regime in the laboratory representation^33^. Moreover, compared with^29^, our scheme reaches the maximal cooling rate *W*_*max*_ > 0.5*λ* by a weaker cooling laser, e.g., approximately Ω/2*π* = 2 MHz, for the lower frequency vibrational mode, e.g., *ω*_*k*_/2*π* = 2 MHz. In contrast, reaching such a cooling rate *W* in^29^ requires *ω*_*k*_/2*π* = 10 MHz and Ω/2*π* = 80 MHz, where the Rabi frequency Ω is linearly proportional to the vibrational frequency *ω*_*k*_. So our scheme can release the demanding experimental requirement of strong laser beams compared with the previous scheme. In addition, the figures also present that cooling the cantilever with *ω*_*k*_/2*π* < 1 MHz is less efficient compared with *ω*_*k*_/2*π* = 2 or 3 MHz, but still working. For example, from Figs 5 and 6, we know that our scheme can cool the cantilever down to 〈*n*〉_*ss*_ ≈ 0.2 with the cooling rate 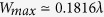 in the case of *ω*_*k*_/2*π* = 0.5 MHz and Ω/2*π* = 2 MHz. In contrast, the schemes in^29^,^33^ can work only in the case of *ω*_*k*_/2*π* ≥ 1 MHz and a larger Rabi frequency (e.g., Ω/2*π* = 5 MHz^29^).

## Discussion

In terms of the experimental parameters reported^34^,^45^,^46^, we may choose following parameter values for our scheme. The NR with a frequency 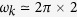 MHz takes a decay rate *γ* = 100 Hz. The laser with wavelength 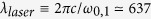 nm and power *P* ≪ 100 *μW* can induce the Rabi frequency Ω = 2*π* × 2 MHz. Then, under the action of the external MFG (~10^7^ T/m^33^,^46^), the NR can be cooled down to its ground state with a mean phonon number 〈*n*〉_*ss*_ < 0.1 and a NV-cantilever coupling 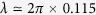 MHz at the temperature *T* = 20 mK.

**Figure 7:**
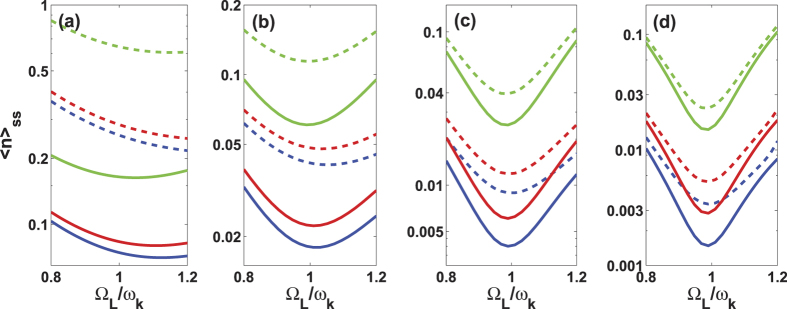
The final average phonon number 〈*n*〉_*ss*_ (in logarithmic scale) versus Ω_*L*_/*ω*_*k*_ for different *ω*_*k*_, where (**a**) *ω*_*k*_/2*π* = 0.5 MHz; (**b**) *ω*_*k*_/2*π* = 1 MHz; (**c**) *ω*_*k*_/2*π* = 2 MHz; (**d**) *ω*_*k*_/2*π* = 3 MHz. In every panel, the solid curves are simulated by equation (9) and the dash curves are the numerical results by the master equation of *H*^*rot*^. The pairs of the curves, from the bottom to top, correspond to Γ_*k*_/2*π* = 0, 1 and 10 Hz, respectively. Other parameters take the values as Ω/2*π* = 2 MHz, Γ/2*π* = 15 MHz, *T* = 20 mK, *λ*/2*π* = 0.115 MHz and Δ = 0.

To check how well our scheme works in a realistic system, we consider below the variation of the cooling effect with respect to the deviation from the work point Ω_*L*_ = *ω*_*k*_ of the Stark-shift gate. Figure 7 presents that the average phonon number 〈*n*〉_*ss*_ changes slightly with *ω*_*k*_, but this change becomes less evident for a larger *ω*_*k*_. Since the work point only maximizes *A*_−_, the fact that 〈*n*〉_*ss*_ is determined by both *A*_−_ and *A*_+_ leads to the onset of the minimal average phonon number deviated from the work point. For a given decay rate Γ_*k*_, the larger *ω*_*k*_ is more beneficial for cooling at the work point, and less sensitive to the deviation from the work point. In summary, our proposed cooling is robust against the experimental imperfection.

We should also assess the influence from the nuclear spin bath in the NV center, which might seriously affect the final average phonon number 〈*n*〉_*ss*_ and the cooling time *t*. To this end, we have considered some concrete values of the parameters, such as 〈*n*〉_*initial*_ = 5, *ω*_*k*_/2*π* = 2 MHz, *λ*/2*π* = 0.115 MHz, Γ/2*π* = 15 MHz, *T* = 20 mK, Ω/2*π* = 2 MHz, Ω_*L*_ = *ω*_*k*_, Δ = 0 and Γ_*k*_/2*π* = 10 Hz. Provided the nuclear spin bath taking the random energy *δ*_*n*_/2*π* ≤ 0.1 MHz (0.5 MHz), the final average phonon number increases from 〈*n*〉_*ss*_ = 0.0399 to 0.0436 (0.1729) and the corresponding cooling takes time from *t* = 39.7 *μ*s to 42.2 *μ*s (97.5 *μ*s). Therefore, suppressing the influence from the nuclear spin bath is very important in order to achieve our cooling scheme. Possible approaches include the dynamic nuclear polarization technology^47^ and the isotopic purification of NV center^48^, which have been widely adopted in the field of spintronics.

In summary, we have studied an efficient cooling of the cantilever vibration by a dynamic Stark-shift gate, in which the carrier transition and the blue-sideband transition can be effectively suppressed when the operation is made around the work point of the Stark-shift gate. We have shown the possibility to cool the low-frequency cantilever down to the vicinity of the ground state using weak cooling lasers. Most of the parameter values are taken from experimental reports. Particularly, our scheme is less stringent experimentally compared to previous schemes, such as with weak laser radiation (i.e., smaller laser power), working for lower-frequency cantilevers (i.e., usually employed cantilevers) and with a feasible MFG ~10^7^ T/m^33^,^46^ (without special requirement for the magnetic field strength). As such our scheme is experimentally relevant and feasible.

Compared to the previous proposal for the trapped ion^32^, our scheme has major differences at following two points. The essential difference is the much larger mass of the cantilever compared to the trapped ion. As a result, in our case the coupling of the motional degrees of freedom of the cantilever to the spin degrees of freedom of the NV center is provided by a strong magnetic field gradient. In contrast, a laser radiation simply achieves this coupling^32^. The second difference lies in different technical requirements. For example, the effective coupling strength Ω_*L*_ is achieved by using a Raman transition in our case. In a word, the dynamical Stark-shift in our case is from the classical-field-assisted MFG, rather than the laser field as in^32^.

Finally, we have to mention that the analytical results we obtained, although not always accurate, have presented a clear relationship of the final average phonon number with the vibrational decay rate and the bath temperature. Moreover, we have also analyzed the limitation of the maximal cooling rate. We argue that our scheme is experimentally timely and would be useful for achieving an efficient cooling of the cantilever vibration using currently available techniques.

## Methods

### Two-photon Raman process

The ground state sublevels 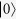 and 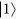 cannot be coupled directly unless the external magnetic field applied is not exactly in parallel with the NV crystal axis. Unfortunately, our model requires the external magnetic field to be applied exactly along the axis of the NV crystal. To solve this problem, we consider an effective coupling between 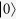 and 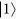 created by a two-photon Raman process, where two additional lasers with large detunings from the 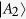 are employed to couple, respectively, 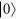 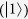 to the exited state 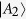 with frequency 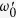 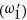 and Rabi frequency 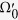 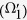. Under the large detuning Δ′ (|Δ′| ≫ |Δ|, 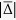) where 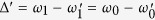, the additional lasers can induce an effective coupling between 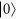 and 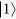 with the coupling intensity 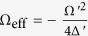. Therefore, the two-photon Raman process can realize the effect of an effective classical field with Ω_*L*_ = Ω_eff_. (See Supplementary Information for details).

### The cooling and heating rates

In what follows, we derive the cooling and heating rates by the non-equilibrium fluctuation-dissipation relation. Using *X* = *x*_0_(*a*^†^ + *a*), we rewrite the interaction Hamiltonian in equation (3) as 
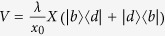
. As a result, the Heisenberg operator *F*(*t*) is given by 
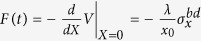
 where we have defined 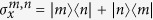 with *m, n* = *A*_2_, *b, d*. The steady state *ρ*_*ss*_ for the NV center can be solved as 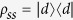 by the Bloch equations of *H*^*rot*^ 49 (see Supplementary Information). When the NV center is in the dark state, the fluctuation spectrum is written as


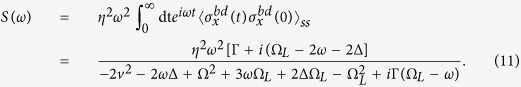


The corresponding heating (cooling) coefficient can be obtained by 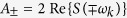.

### The numerical simulation

To check the analytical results, we simulate the dynamical process by the master equation of the density matrix *ρ*. The master equation for the density matrix *ρ* in the Lindblad form^26^,^29^ is given by


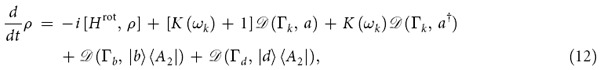


where 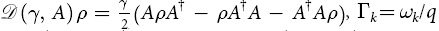, Γ_*k*_ = *ω*_*k*_/*q* and 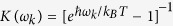 with the cantilever quality *q* and the environmental temperature *T*.

## Additional Information

**How to cite this article**: Yan, L. *et al.* Fast optical cooling of a nanomechanical cantilever by a dynamical Stark-shift gate. *Sci. Rep.* **5**, 14977; doi: 10.1038/srep14977 (2015).

## Supplementary Material

Supplementary Information
