# Supplementary material for: Fast optical cooling of a nanomechanical cantilever by a dynamical Stark-shift gate
Source: Sci Rep. 2015 Oct 12;5:14977. doi: 10.1038/srep14977 (PMC4601024; doi:10.1038/srep14977)
Supplement: Supplementary Information [file srep14977-s1.pdf]

Supplementary Information for "Fast optical cooling of a nanomechanical cantilever by a dynamical Stark-shift gate"

Leilei Yan, Jian-Qi Zhang, Shuo Zhang and Mang Feng

Two-photon Raman process. The ground state sublevels $|0\rangle$ and $|1\rangle$ cannot be coupled directly unless the external magnetic field applied is of an angle with respect to the NV crystal axis. Unfortunately, our model requires the external magnetic field to be applied exactly along the axis of the NV crystal. As a result, we have to consider the effective coupling between $|0\rangle$ and $|1\rangle$ by a two-photon Raman process, where two additional lasers with large detunings from the $|A_2\rangle$ are employed to couple, respectively, $|0\rangle$ and $|1\rangle$ to the exited state $|A_2\rangle$ with frequency ω_0' (ω_1') and Rabi frequency Ω_0' (Ω_1'). In this context, the original model for cooling plotted in Supplementary Figure 1(a) includes other two laser fields applied to drive the transition from the ground state $|0\rangle$ ($|1\rangle$) to the excited one $|A_2\rangle$ with frequency $\bar{\omega}_0$ ($\bar{\omega}_1$) and Rabi frequency $\bar{\Omega}_0$ ($\bar{\Omega}_1$). Please note that, for the same parameters, we define different symbols from in the main text due to slight difference of parameter values after the two-photon Raman process is applied (see equation (4) below).

Thus our system is governed by the Hamiltonian

$$\begin{aligned} H \;=\; & \omega_k a^\dagger a + \omega_A |A_2\rangle\langle A_2| + g_e\mu_B B(0)(|1\rangle\langle 1| - |0\rangle\langle 0|) \\ & + \frac{\bar{\Omega}}{2}(|A_2\rangle\langle 1| e^{-i\bar{\omega}_1 t} + |A_2\rangle\langle 0| e^{-i\bar{\omega}_0 t} + h.c.) \\ & + \frac{\Omega'}{2}(|A_2\rangle\langle 1| e^{-i\omega_1' t} + |A_2\rangle\langle 0| e^{-i\omega_0' t} + h.c.) \\ & + \lambda(|1\rangle\langle 1| - |0\rangle\langle 0|)(a^\dagger + a), \end{aligned} \tag{1}$$

where $\bar{\Omega} = \bar{\Omega}_0 = \bar{\Omega}_1$ and $\Omega' = \Omega_0' = \Omega_1'$.

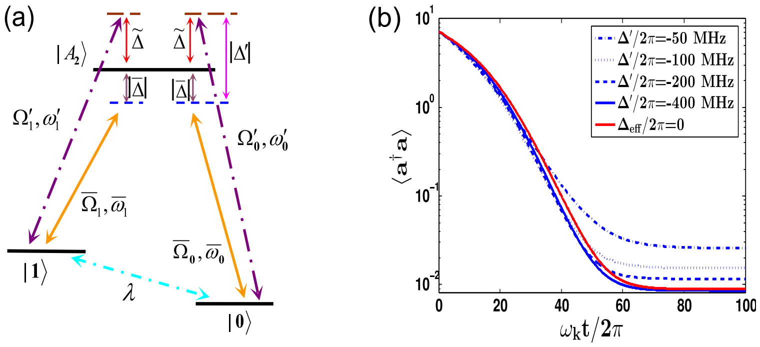

Supplementary Figure 1: (a) Schematic of the scheme, where the dashed-dotted double arrows represent the two-photon Raman process, and the solid double arrows represent the same lasers as plotted in figure 1(b) of the main text; (b) The average phonon number $\langle a^\dagger a\rangle$ versus the dimensionless evolution time t. The blue curves is calculated by the master equation of equation (2) and the red line is plotted by the master equation of equation (3), where $\Delta_{eff} = 0$, $\bar{\Delta} = \frac{3\Omega'^2}{4\Delta'} = -\frac{3\omega_k}{2}$ and $\Omega' = \sqrt{-2\Delta'\omega_k}$. Other parameter values are $\omega_k/2\pi = 2$ MHz, $\lambda/2\pi = 0.115$ MHz, $\Gamma/2\pi = 15$ MHz and $\bar{\Omega}/2\pi = 2$ MHz.

In the rotating frame with $R \equiv \bar{\omega}_1 |1\rangle\langle 1| + \bar{\omega}_0 |0\rangle\langle 0|$, the whole system is described as

$$\begin{aligned} H \;=\; & \omega_k a^\dagger a - \bar{\Delta} |A_2\rangle\langle A_2| + \frac{\bar{\Omega}}{2}(|A_2\rangle\langle 1| + |A_2\rangle\langle 0| + h.c.) \\ & + \frac{\Omega'}{2}(|A_2\rangle\langle 1| e^{i\Delta' t} + |A_2\rangle\langle 0| e^{i\Delta' t} + h.c.) \\ & + \lambda(|1\rangle\langle 1| - |0\rangle\langle 0|)(a^\dagger + a), \end{aligned} \tag{2}$$

where $\bar{\Delta} \equiv g_e\mu_B B(0) + \bar{\omega}_1 - \omega_A = -g_e\mu_B B(0) + \bar{\omega}_0 - \omega_A$ and $\Delta' = \omega_1 - \omega_1' = \omega_0 - \omega_0'$ with $\tilde{\Delta} \equiv g_e\mu_B B(0) + \omega_1' - \omega_A = -g_e\mu_B B(0) + \omega_0' - \omega_A = \bar{\Delta} - \Delta'$. Under the large detuning Δ' and utilizing the method proposed in [1], we obtain an effective Hamiltonian

$$\begin{aligned} H_{eff} \;=\; & \omega_k a^\dagger a - \Delta_{eff} |A_2\rangle\langle A_2| + \frac{\bar{\Omega}}{2}(|A_2\rangle\langle 1| + |A_2\rangle\langle 0| + h.c.) \\ & - \frac{\Omega'^2}{4\Delta'}(|0\rangle\langle 1| + |1\rangle\langle 0|) + \lambda(|1\rangle\langle 1| - |0\rangle\langle 0|)(a^\dagger + a), \end{aligned} \tag{3}$$

where $\Delta_{eff} = \bar{\Delta} - \frac{3\Omega'^2}{4\Delta'}$ and $\Delta' < 0$. Note that the energy shifts caused by the Raman process have been included in this effective Hamiltonian. In the case of $-\frac{\Omega'^2}{4\Delta'} = \frac{\Omega_L}{2} = \frac{\omega_k}{2}$, i.e. $\Omega' = \sqrt{-2\Delta'\omega_k}$, equation (3) here is unitarily equivalent to equation (2) plus equation (3) in the main text.

The corresponding parameters in equation (2) of the main text and in above equations (1, 2, 3) are as follows,

$$\begin{aligned}
\Delta &= \bar{\Delta} - \tfrac{3\Omega'^2}{4\Delta'}, \\
\omega_1 &= \bar{\omega}_1' - \tfrac{3\Omega'^2}{4\Delta'}, \ \omega_2 = \bar{\omega}_2' - \tfrac{3\Omega'^2}{4\Delta'}, \\
\Omega_L &= -\tfrac{\Omega'^2}{2\Delta'}, \quad \Omega = \bar{\Omega}.
\end{aligned} \tag{4}$$

To justify the effective Hamiltonian in equation (2), we numerically compare equation (2) with equation (3) in Supplementary Figure 1(b), which shows a good fitting in the case of $\Delta'/2\pi = -400$ MHz.

The cooling and heating rates. In what follows, we derive the cooling and heating rates by the non-equilibrium fluctuation-dissipation relation. Using $X = x_0(a^\dagger + a)$, we rewrite the interaction Hamiltonian in equation (3) in the main text as

$$V = \lambda(|b\rangle\langle d| + |d\rangle\langle b|)(a^\dagger + a) = \frac{\lambda}{x_0}X(|b\rangle\langle d| + |d\rangle\langle b|). \tag{5}$$

As a result, the Heisenberg operator $F(t)$ is given by

$$F(t) = -\frac{d}{dX}V\,|_{X=0} = -\frac{\lambda}{x_0}(|b\rangle\langle d| + |d\rangle\langle b|) = -\frac{\lambda}{x_0}\sigma_x^{bd}, \tag{6}$$

where we have defined $\sigma_x^{m,n} = |m\rangle\langle n| + |n\rangle\langle m|$ with $m, n = A_2, b, d$.

The steady state ρ_{ss} for the NV center can be obtained from following Bloch equations of H^{rot} [2],

$$\begin{aligned}
\tfrac{d\langle\rho^{bb}\rangle}{dt} &= -\tfrac{\sqrt{2}\Omega}{2}\langle\sigma_y^{A_2,b}\rangle + \Gamma_b(1 - \langle\rho^{bb}\rangle - \langle\rho^{dd}\rangle), \\
\tfrac{d\langle\rho^{dd}\rangle}{dt} &= \Gamma_d(1 - \langle\rho^{bb}\rangle - \langle\rho^{dd}\rangle), \\
\tfrac{d\langle\sigma_x^{bd}\rangle}{dt} &= -\tfrac{\sqrt{2}\Omega_0}{2}\langle\sigma_y^{A_2,d}\rangle - \Omega_L\langle\sigma_y^{b,d}\rangle, \\
\tfrac{d\langle\sigma_y^{bd}\rangle}{dt} &= \tfrac{\sqrt{2}\Omega}{2}\langle\sigma_x^{A_2,d}\rangle + \Omega_L\langle\sigma_x^{b,d}\rangle, \\
\tfrac{d\langle\sigma_x^{A_2,b}\rangle}{dt} &= -\tfrac{\Gamma}{2}\langle\sigma_x^{A_2,b}\rangle + \Delta\langle\sigma_y^{A_2,b}\rangle + \tfrac{\Omega_L}{2}\langle\sigma_y^{A_2,b}\rangle, \\
\tfrac{d\langle\sigma_y^{A_2,b}\rangle}{dt} &= -\tfrac{\Gamma}{2}\langle\sigma_y^{A_2,b}\rangle + \sqrt{2}\Omega(2\langle\rho^{bb}\rangle + \langle\rho^{dd}\rangle - 1) - \Delta\langle\sigma_x^{A_2,b}\rangle - \tfrac{\Omega_L}{2}\langle\sigma_x^{A_2,b}\rangle, \\
\tfrac{d\langle\sigma_x^{A_2,d}\rangle}{dt} &= -\tfrac{\Gamma}{2}\langle\sigma_x^{A_2,d}\rangle - \tfrac{\sqrt{2}\Omega}{2}\langle\sigma_y^{bd}\rangle + \Delta\langle\sigma_y^{A_2,d}\rangle - \tfrac{\Omega_L}{2}\langle\sigma_y^{A_2,d}\rangle, \\
\tfrac{d\langle\sigma_y^{A_2,d}\rangle}{dt} &= -\tfrac{\Gamma}{2}\langle\sigma_y^{A_2,d}\rangle + \tfrac{\sqrt{2}\Omega}{2}\langle\sigma_x^{bd}\rangle - \Delta\langle\sigma_x^{A_2,d}\rangle + \tfrac{\Omega_L}{2}\langle\sigma_x^{A_2,d}\rangle,
\end{aligned} \tag{7}$$

where $\Gamma_d(=\Gamma_b) = (\gamma_0 + \gamma_1)/2$ is the decay rate from the excited state $|A_2\rangle$ to the state $|d\rangle$ ($|b\rangle$), $\Gamma = \gamma_0 + \gamma_1$ is the total decay rate with γ_1 and γ_0 being the decay rates, respectively, from the excited state $|A_2\rangle$ to the states $|1\rangle$ and $|0\rangle$, $\rho^{bb} = |b\rangle\langle b|$, $\rho^{dd} = |d\rangle\langle d|$ and $\sigma_y^{m,n} = -i(|m\rangle\langle n| - |n\rangle\langle m|)$. The steady state for these Bloch equations can be solved by making the left side of equation 7 equal to zero (i.e., $\frac{d\langle\cdots\rangle}{dt} = 0$) and it is $\rho_{ss} = \rho^{dd}$, which means a dark state regarding the NV center.

When the NV center is in the dark state, the fluctuation spectrum is written as

$$S(\omega) = \eta^2\omega^2\int_0^\infty \mathrm{d}t e^{i\omega t}\langle\sigma_x^{bd}(t)\sigma_x^{bd}(0)\rangle_{ss}, \tag{8}$$

where $\eta = \lambda/\omega$. According to the quantum regression theorem [2], the differential equations of the correlation functions

can be written as

$$
\begin{aligned}
\frac{d\langle\rho^{bb}(t)\sigma_x^{bd}(0)\rangle_{ss}}{dt} &= -\frac{\sqrt{2}\Omega}{2}\langle\sigma_y^{A_2,b}(t)\sigma_x^{bd}(0)\rangle_{ss} + \Gamma_b(\langle\sigma_x^{bd}(0)\rangle_{ss} - \langle\rho^{bb}(t)\sigma_x^{bd}(0)\rangle_{ss} - \langle\rho^{dd}(t)\sigma_x^{bd}(0)\rangle_{ss}),\\
\frac{d\langle\rho^{dd}(t)\sigma_x^{bd}(0)\rangle_{ss}}{dt} &= \Gamma_d(1 - \langle\rho^{bb}(t)\sigma_x^{bd}(0)\rangle_{ss} - \langle\rho^{dd}(t)\sigma_x^{bd}(0)\rangle_{ss}),\\
\frac{d\langle\sigma_x^{bd}(t)\sigma_x^{bd}(0)\rangle_{ss}}{dt} &= -\frac{\sqrt{2}\Omega}{2}\langle\sigma_y^{A_2,d}(t)\sigma_x^{bd}(0)\rangle_{ss} - \Omega_L\langle\sigma_y^{b,d}(t)\sigma_x^{bd}(0)\rangle_{ss},\\
\frac{d\langle\sigma_y^{bd}(t)\sigma_x^{bd}(0)\rangle_{ss}}{dt} &= \frac{\sqrt{2}\Omega}{2}\langle\sigma_x^{A_2,d}(t)\sigma_x^{bd}(0)\rangle_{ss} + \Omega_L\langle\sigma_x^{b,d}(t)\sigma_x^{bd}(0)\rangle_{ss},\\
\frac{d\langle\sigma_x^{A_2,b}(t)\sigma_x^{bd}(0)\rangle_{ss}}{dt} &= -\frac{\Gamma}{2}\langle\sigma_x^{A_2,b}(t)\sigma_x^{bd}(0)\rangle_{ss} + (\Delta + \frac{\Omega_L}{2})\langle\sigma_y^{A_2,b}(t)\sigma_x^{bd}(0)\rangle_{ss},\\
\frac{d\langle\sigma_y^{A_2,b}(t)\sigma_x^{bd}(0)\rangle_{ss}}{dt} &= -\frac{\Gamma}{2}\langle\sigma_y^{A_2,b}(t)\sigma_x^{bd}(0)\rangle_{ss} + \sqrt{2}\Omega(2\langle\rho^{bb}(t)\sigma_x^{bd}(0)\rangle_{ss} + \langle\rho^{dd}(t)\sigma_x^{bd}(0)\rangle_{ss}\\
&\quad - \langle\sigma_x^{bd}(0)\rangle_{ss}) - (\Delta + \frac{\Omega_L}{2})\langle\sigma_x^{A_2,b}(t)\sigma_x^{bd}(0)\rangle_{ss},\\
\frac{d\langle\sigma_x^{A_2,d}(t)\sigma_x^{bd}(0)\rangle_{ss}}{dt} &= -\frac{\Gamma}{2}\langle\sigma_x^{A_2,d}(t)\sigma_x^{bd}(0)\rangle_{ss} - \frac{\sqrt{2}\Omega}{2}\langle\sigma_y^{bd}(t)\sigma_x^{bd}(0)\rangle_{ss}\\
&\quad + (\Delta - \frac{\Omega_L}{2})\langle\sigma_y^{A_2,d}(t)\sigma_x^{bd}(0)\rangle_{ss},\\
\frac{d\langle\sigma_y^{A_2,d}(t)\sigma_x^{bd}(0)\rangle_{ss}}{dt} &= -\frac{\Gamma}{2}\langle\sigma_y^{A_2,d}(t)\sigma_x^{bd}(0)\rangle_{ss} + \frac{\sqrt{2}\Omega}{2}\langle\sigma_x^{bd}(t)\sigma_x^{bd}(0)\rangle_{ss} - (\Delta - \frac{\Omega_L}{2})\langle\sigma_x^{A_2,d}(t)\sigma_x^{bd}(0)\rangle_{ss}.
\end{aligned}
$$

Defining a Fourier transformation

$$f(t) \rightleftharpoons \mathfrak{F}(\omega) = \int_0^\infty \mathrm{d}t e^{i\omega t} f(t), \tag{9}$$

we obtain

$$\frac{df(t)}{dt} \rightleftharpoons -f(0) - i\omega\mathfrak{F}(\omega). \tag{10}$$

Applying the transformation to the differential equations and solving the corresponding equations, we have

$$S(\omega) = \eta^2\omega^2 \int_0^\infty \mathrm{d}t e^{i\omega t}\langle\sigma_x^{bd}(t)\sigma_x^{bd}(0)\rangle_{ss} = \frac{\eta^2\omega^2[\Gamma + i(\Omega_L - 2\omega - 2\Delta)]}{-2v^2 - 2\omega\Delta + \Omega^2 + 3\omega\Omega_L + 2\Delta\Omega_L - \Omega_L^2 + i\Gamma(\Omega_L - \omega)}, \tag{11}$$

and the corresponding heating (cooling) coefficient A_+ (A_-)

$$
\begin{aligned}
A_\pm &= 2\mathrm{Re}\{S(\mp\omega_k)\}\\
&= \frac{2\Gamma\lambda^2\Omega^2}{[\Omega^2 + (\mp\omega_k - \Omega_L)(\pm 2\omega_k - 2\Delta + \Omega_L)]^2 + \Gamma^2(\mp\omega_k - \Omega_L)^2}.
\end{aligned} \tag{12}
$$

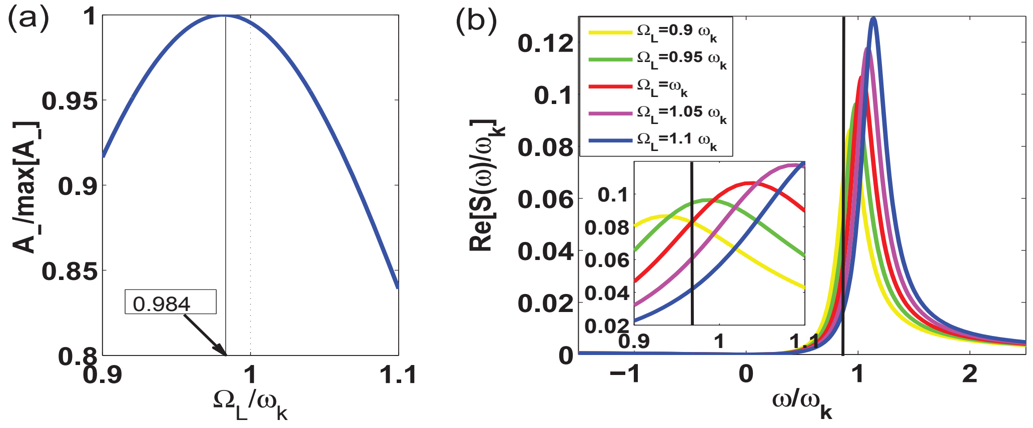

Supplementary Figure 2: (a) The renormalized cooling coefficient A_- vs the dimensionless Rabi frequency Ω_L/ω_k, where the vertical solid line indicates the maximum at Ω_L/ω_k =0.984 obtain by derivative of A_- with respect to Ω_L and the vertical dashed line points out the maximum obtain by the work point $\Omega_L/\omega_k = 1$ of the Stark-shift gate. (b) The real part of the fluctuation spectra in our scheme, where $\omega/\omega_k = 1(-1)$ means the red(blue)-detuning and $\omega/\omega_k = 0$ means the carrier transition. The inset is a zoom-in plot of the cooling coefficient A_-. We take $\Gamma/2\pi = 15$

MHz, $\Delta = 0$, $\Omega/2\pi = 2$ MHz and $\lambda = 0.115\omega_k$.

We discuss by equation (12) whether the cooling efficiency can be optimal at the work point $\Omega_L/\omega_k = 1$ of the Stark-shift gate. A straightforward evaluation can be done by $\frac{\partial}{\partial\Omega_L}A_- = 0$. As comparison in Supplementary Figure 2(a), the optimal cooling efficiency occurs at Ω_L/ω_k =0.984, slightly different from the work point of the Stark-shift gate. The difference is resulted from the rotating wave approximation we made for obtaining the work point of the Stark-shift gate and also from the energy correction caused by the external light fields. Specifically, if we consider a second-order approximation of Γ under the condition $\Gamma \gg \max[\Delta, \Omega, \omega]$, we may have an analytical expression of the difference $\Omega_L - \omega_k \propto (\omega_k + 2\Delta)[5(\omega_k + 2\Delta)^2 - 16\Omega^2]/\Gamma^2$. Since the difference is tiny, we may still employ the work point of the Stark-shift gate as the approximately optimal cooling point, which is physically clear and easily understood.

To be more clarified, we plot $\mathrm{Re}[S(\omega)]$ for various Ω_L in Supplementary Figure 2(b). In fact, we know from equation (12) that, different from the fluctuation spectra of EIT obtained in Ref. [3], the Stark-shift fluctuation spectra maximize at different values conditional on Rabi frequencies Ω, Ω_L and detuning Δ.

---

[1] James, D. F. V. & Jerke, J. Effective Hamiltonian theory and its applications in quantum information. *Can. J. Phys.* **85**, 625-632 (2007).

[2] Cirac, J. I., Blatt, R. & Zoller, P. Laser cooling of trapped ions in a standing wave. *Phys. Rev. A* **46**, 2668 (1992).

[3] Zhang, J. Q., Zhang, S., Zou, J. H., Chen, L., Yang, W., Li, Y. & Feng, M. Fast optical cooling of nanomechanical cantilever with the dynamical Zeeman effect *Optics Express* **21**, 029695 (2013).
